# Using a Combination of ECAP and HE Processes to Produce Isotropic Ultrafine-Grained Titanium

**DOI:** 10.3390/ma18225194

**Published:** 2025-11-15

**Authors:** Mariusz Kulczyk, Jacek Skiba, Sylwia Przybysz-Gloc, Łukasz Maj, Jakub Kawałko, Monika Skorupska

**Affiliations:** 1 Institute of High Pressure Physics, Polish Academy of Sciences (Unipress), ul. Sokolowska 29, 01-142 Warszawa, Poland; skiba@unipress.waw.pl (J.S.); sylwia@unipress.waw.pl (S.P.-G.); monikaw@unipress.waw.pl (M.S.); 2 Institute of Metallurgy and Materials Science, Polish Academy of Sciences, Reymonta 25, 30-059 Krakow, Poland; l.maj@imim.pl; 3 Academic Centre for Materials and Nanotechnology, AGH University of Kraków, Al. A. Mickiewicza 30, 30-059 Krakow, Poland; kubaka@agh.edu.pl

**Keywords:** titanium grade 2, equal-channel angular pressing (ECAP), hydrostatic extrusion (HE), microstructural anisotropy, anisotropy of mechanical properties, severe plastic deformation (SPD)

## Abstract

This paper proposes a complex plastic forming process for grade 2 titanium using a combination of severe plastic deformation techniques, equal-channel angular pressing (ECAP) and hydrostatic extrusion (HE). The aim of the combination of these methods is to reduce the strength of the phenomenon of microstructural anisotropy and the resulting anisotropy of mechanical properties characteristic of the HE process. Two routes of plastic deformation (HE and ECAP + HE) were compared using the same total strain rate of ε ~ 3.5. Results: The analysis of mechanical properties and microstructure studies using TEM and SEM/EBSD techniques showed that it is possible to obtain large microstructure refinement of titanium with almost identical mechanical properties via both studied techniques. In addition, this also leads to a significant improvement in the strength of UTS by ~ 1000 MPa, YS by ~ 945 MPa and ductility (E) by ~ 25%. These findings indicate that applying a sequential ECAP + HE strategy can effectively reduce anisotropy and improve the overall performance of grade 2 titanium.

## 1. Introduction

Titanium is one of the most popular materials used for medical implants. This is due to its inertness in the human body environment, biocompatibility, ability to osseointegrate with bone and lack of magnetism.

Due to the relatively low mechanical strength and wear resistance of pure titanium, alloying additions such as aluminum, vanadium or niobium are commonly used. Studies conducted on the effects of these additives on the behavior of the material in the human body show that they are harmful to health, as they are toxic and carcinogenic [1,2,3]. Utilization of commercially pure titanium may be possible by refining its microstructure, leading to an increase in strength without changing the chemical composition. The most effective methods of microstructure refinement are severe plastic deformation methods, in which microstructural defects are accumulated in the material, leading to the transformation of medium-sized grains to the nanocrystalline or ultrafine level.

The first studies were carried out many years ago. The most popular methods used include equal-channel angular pressing (ECAP), high pressure torsion (HPT) or cold/hot rolling and various combinations of these methods [4,5,6]. In many cases, strong refinement of the microstructure has been reported, even down to the nanometer level, and drastic improvements in strength allow the materials to reach levels close to commercially used titanium alloys like Ti-6Al-4V. Despite this, these types of materials have not been commercialized on a widespread scale and are not currently widely available on the market.

One reason for this is the heterogeneity of the materials produced by this type of method, and another is the geometry of the final product of the plastic deformation [7,8]. Commercial implant manufacturing requires semi-finished products in the form of long rods that can be machined on numerical machines. This is impossible using such large plastic deformation techniques as ECAP or HPT. Hence, researchers are working on modifying the methods used so far by designing new solutions to enable continuous plastic deformation processes. An example of this is the continuous ECAP (ECAP-Conform) process, which allows for the production of higher volume intermediates [5,9]. However, such processes require much higher forces, often elevated temperatures and do not lead to such effective improvement of mechanical properties. The poor surface quality of manufactured intermediates and the inhomogeneity of properties require the use of additional post-deformation processing such as drawing [10].

An alternative solution is to use the hydrostatic extrusion process. It is a globally unique method of large plastic deformation, using very high hydrostatic pressure to generate deformation of the material [11]. High hydrostatic pressure protects against the formation of microcracks in the material and inhibits the propagation of existing microcracks. This makes it possible to realize plastic deformation processes with high real deformation in a single extrusion operation. The effectiveness of using the hydrostatic extrusion method to break up the microstructure and improve the mechanical properties of materials has been confirmed in the literature for many metals and alloys. Studies of this type have been conducted so far for both easily deformable materials, such as aluminum or aluminum alloys, copper and copper alloys and zinc alloys, and materials that are difficult to deform, such as austenitic steels, brittle materials such as magnesium and magnesium alloys, materials in powder form and even plastics [12,13,14,15,16,17,18].

The first experiments using the hydrostatic extrusion process for plastic deformation of titanium were extensively reported in as early as 2008 [19]. In this work, the actual deformation of ~ 5.5 was accumulated in the produced semi-finished products, obtaining an average grain size of deq ~ 50 nm with an improvement in mechanical properties, e.g., yield strength (YS) and tensile strength (UTS), by nearly 150% compared to the starting material, i.e., YS ~ 1250 MPa and UTS ~ 1300 MPa. The material showed high homogeneity over the cross-section of the tested bars and had a good yield strength (elongation ~ 8%). However, the tests carried out required 19 passes in the hydrostatic extrusion process, and the resulting product had a diameter of only 3 mm and was characterized by very poor surface quality, which was related to the lack of pressure stability during the hydrostatic extrusion process. A much improved process for hydrostatic extrusion of titanium was presented in a paper published in 2015 [20]. Optimization of the parameters of the hydrostatic extrusion process allowed the achievement of a true deformation of ε ~ 3 in just two passes, and the use of a post-deformation rotary forging process led to long products with very good surface quality, which made it possible to further shape the material using precision machining methods. Semi-finished products in the form of long rods with a diameter of 9 mm were obtained, and the average grain size in the tested material was deq ~ 100 nm. UTS strengths of more than 1100 MPa were demonstrated, as well as high reproducibility of results in accurate mechanical measurements realized along the length of the produced bars.

Prototypes of medical implants were manufactured from such prepared materials in cooperation with a company specializing in such products. Subsequent unpublished research, carried out by the implant manufacturer, consisted of dynamic bending tests of long specimens in a four-point arrangement which showed very poor fatigue resistance of the fabricated prototypes compared to commonly used Ti-6Al-4V alloys. According to these studies, this was related to the phenomenon of strong microstructural anisotropy appearing in titanium after the hydrostatic extrusion process. This phenomenon has already been presented earlier, during mechanical tests carried out on micro-samples [21]. Observations of the microstructure of titanium after the HE process was performed on the longitudinal cross-section showed a strong orientation of the microstructure, according to the direction of extrusion. While the grains were evenly aligned on the cross-section, long grains were observed on the longitudinal section in the form of strongly oriented bands. Mechanical tests in which the force was applied perpendicular to the direction of the fibers showed significantly smaller yield strength (YS) values compared to measurements where the force was in line with the direction of the fibers. In the same publication, micro-punching tests (SPTs) were presented which showed significant differences in the nature of cracking on the two cross-sections, where pronounced cracks formed along the elongated grains were observed.

In this publication, the authors present a plastic processing technology for reducing the phenomenon of microstructural anisotropy in titanium after a hydrostatic extrusion process. For this purpose, a combination of ECAP and HE processes was used for the first time for pure titanium. The study assumed that the ECAP method could be used to pre-fine the microstructure, significantly reducing the effects of its anisotropy after the subsequent extrusion process compared to the application of the extrusion process alone on coarse-grained material. The results presented in the paper were verified by meticulous mechanical tests performed on micro specimens and microstructural studies using TEM and EBSD techniques. In contrast to earlier ECAP + HE studies [22], where deformation was performed at elevated temperature or applied to other alloys and higher-purity titanium grades [21], the present work introduces a fully cold route (two ECAP passes in a 120° die with 180° rotation) followed by room-temperature cumulative HE (ε ≈ 3.5) applied to commercially pure Ti grade 2 rods (φ 5 mm). This approach enables the formation of nearly isotropic mechanical properties, verified by overlapping tensile curves and corroborated by TEM and EBSD analyses. To the best of our knowledge, such a quantified demonstration of near-isotropy in cp-Ti rods produced by cold ECAP (120°, two passes) + HE has not been previously reported.

## 2. Materials and Methods

The material tested was grade 2 pure titanium conforming to standards in the form of 30 mm diameter rods. The chemical composition of the initial cp-Ti grade 2 used in this study was taken from the material certificate provided by the supplier of the purchased rods. The certified composition, is listed in Table 1.

In order to eliminate the technological past of the rods and to obtain a fully isotropic initial material for actual extrusion, the titanium was subjected to annealing at 700 °C for 2 h with cooling in air. In order to determine the microstructural isotropy in the initial state, the average equivalent grain size (deq), defined as the diameter of a circle with an area equal to that of a given grain, was determined. The grain elongation coefficients, αPD in the transverse direction and αLD in the longitudinal direction, defined as the ratio of the average maximum diameter to the equivalent diameter, were also determined on both sections. The material was subjected to plastic processing in two different ways:

(1) The process of cumulative four-stage hydrostatic extrusion (HE) of a 29 mm diameter rod with a true strain ε ~ 3.5 for a final diameter of 5 mm.
(2) A combination of ECAP and HE processes where two ECAP passes were applied on a 120° chamber with 180° rotation between subsequent passes for a 15 mm diameter specimen and a cumulative HE process in three stages for a final diameter of 5 mm with a cumulative true strain of ε ~ 3.5.

**Figure 1:**
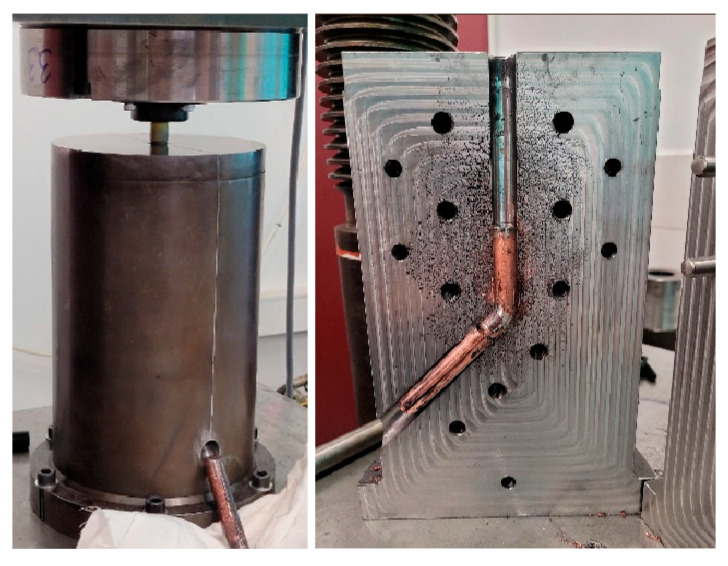
The 120° ECAP process stand for plastic deformation of titanium, IHPP PAS.

In order to be able to compare the results obtained in both cases, the same summed value of true strain was used. To carry out the ECAP process of cold titanium, a special ECAP split chamber with a corner angle of 120° was designed (Figure 1).

**Figure 2:**
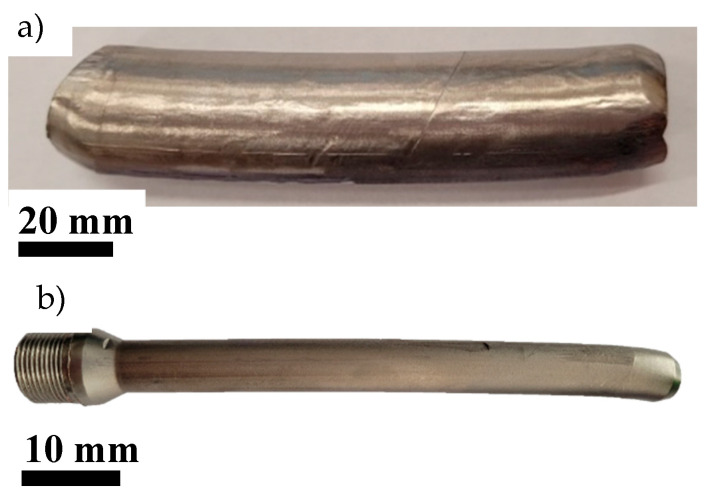
Examples of titanium samples after cold ECAP process (**a**) and after the combined ECAP + HE process with total true strain ε ≈ 3.5 (**b**). The arrows indicate the extrusion direction.

The geometry of the process was optimized to obtain solid products, free of cracks, which is currently the subject of a patent. Details of the two technological processes are shown in Table 2, and an example product in the form of a solid titanium sample after two passes of the ECAP process is shown in Figure 2.

Hydrostatic extrusion processes were carried out at room temperature, using dies with a double angle of α = 45°. HE process stations designed and constructed at IHPP PAS were used, operating in a pressure range up to 2.5 GPa. Mechanical tests after both combinations of plastic deformation processes were carried out were performed using a Inspect Duo 5-M universal testing machine (Hegewald und Peschke MPT GmbH, Nossen, Germany), which allows testing of micro-samples. The machine was equipped with a 5 kN force sensor and a non-contact extensometer for measuring elongation with a resolution of 1 µm. Five micro-samples were prepared from both the longitudinal and transverse sections relative to the extrusion direction. The samples were machined from titanium rods, with a final diameter of 5 mm obtained after the plastic deformation processes. The sample had a flat profile with a measuring section width of 0.8 mm, a measuring length of 2.86 mm and a thickness of 0.6 mm. The transition between the measuring section and the heads was shaped with a radius of R = 0.40 mm at a head angle of 80°. The samples were cut by electro-discharge machining (EDM) to ensure precise geometry and to avoid thermal or mechanical deformation of the surface layer. The specimen axes were oriented either parallel (longitudinal samples) or perpendicular (transverse samples) to the extrusion direction. Static tensile tests were carried out at a speed of 0.02 s^−1^. The results were summarized based on average values calculated for three measurements each time. Analysis of the microstructure of titanium was carried out using light microscopy, transmission electron microscopy (TEM) and scanning microscopy (SEM) techniques. For each of the techniques used, studies were conducted both cross-sectional and longitudinal to the extrusion direction. Light microscopy, implemented using a Nikon Eclipse LV150N (Nikon, Tokyo, Japan) microscope, was used to analyze the microstructure of titanium in its initial state before the deformation process. Samples were ground and polished on SiC papers with gradations up to 2000 and then polished on diamond and oxide slurries. Kroll’s reagent was used to etch the microstructure. TEM studies were carried out using an FEI Tecnai G2 SuperTWIN 200kV FEG (FEI, Eindhoven, The Netherlands) transmission electron microscope equipped with an SIS MegaView III CCD camera (EMSIS, Münster, Germany) for microstructure observations in bright-field mode (TEM/BF) and selected area electron diffraction patterns in parallel beam mode (SAED). Preparation of the TEM samples in the form of thin lamellae (<100 nm thick), transparent to the electron beam in the TEM microscope, was carried out by electrolytic polishing using Struers TenuPol5 and Struers A3 electrolyte (Struers, Copenhagen, Denmark) at ~5 °C. The average grain size after the large plastic deformation processes was determined based on image analysis using Micrometer software v.1.0 [23] and determination of the average equivalent grain size (deq). SEM studies were carried out using a Versa 3D FEG microscope (FEI, Brno, Czech Republic) equipped with Symmetry S2 EBSD (electron backscatter diffraction) camera (Oxford Instruments Nanoanalysis, High Wycombe, UK). Samples were polished on SiC papers with variable gradation up to 2000 and then on oxide suspensions. In the final stage of preparation, etching with Kroll’s reagent was used alternately with polishing with a 1:1 mixture of colloidal silica (OPS) with 30% hydrogen peroxide. After obtaining a mirror-smooth surface of the scrap, the samples were placed in an SEM chamber, and EBSD observations were made. While EBSD maps were recorded, individual diffraction images were saved for later offline analysis using a pattern matching procedure. In this procedure, the algorithm compares experimental diffraction images with images generated in dynamic simulations for the indexed phase (crystal structure). The pattern matching method, together with indexing refinement, allows for a significant improvement in the solvability of EBSD maps, reducing the number of unindexed points. To improve the indexing rate and accuracy of EBSD data, the experimental diffraction patterns were recorded and subsequently used in post-processing procedures involving dynamic template matching and orientation refinement based on simulated diffraction patterns in Oxford Aztec Crystal 3.2 software. Additional data analysis of acquired EBSD maps was performed in Matlab R2024b with MTEX5.0.9 toolbox [24,25].

## 3. Results and Discussion

### 3.1. Mechanical Properties

**Figure 3:**
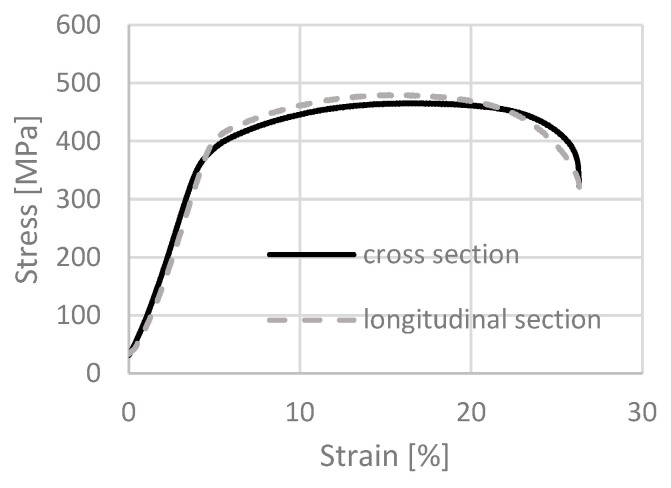
Tensile curves of micro specimens for titanium in the initial state after the annealing process at 700 °C for 2 h for both test directions.

The material in the initial state was characterized by ultimate tensile strength (UTS) = 587 MPa (±12), yield strength (YS) = 491 MPa (±10) and elongation to fracture (E) = 29.5% (±6). The strength characteristics after the annealing process at 700 °C for 2 h are shown in Figure 3.

The curves for the two output directions of the bar practically overlap. The ultimate tensile strength (UTS) resulted in an average UTS = 473 MPa (±6) for the cross-section and 461 MPa (±7) for the longitudinal section. The yield strength (YS) = 389 MPa (±5) and YS = 377 MPa (±6), respectively, and the average values of elongation to fracture (E) = 27 (±4) on the transverse section and E = 28 (±2) on the longitudinal section. A material with such properties was considered by the authors as a reference isotropic initial state. The results confirm that the applied annealing conditions (700 °C/2 h) were sufficient to produce a fully recrystallized and isotropic microstructure, which served as a reliable baseline for evaluating the effects of severe plastic deformation. After plastic deformation using both combinations, i.e., cumulative hydrostatic extrusion with total actual strain ε ~ 3.5 and a combination of the processes of squeezing through an equal channel angular pressing and hydrostatic extrusion with similar values of total strain ε ~ 3.5, comparative tests of mechanical properties on micro-samples were carried out. The tests were implemented on both the longitudinal and transverse sections.

**Figure 4:**
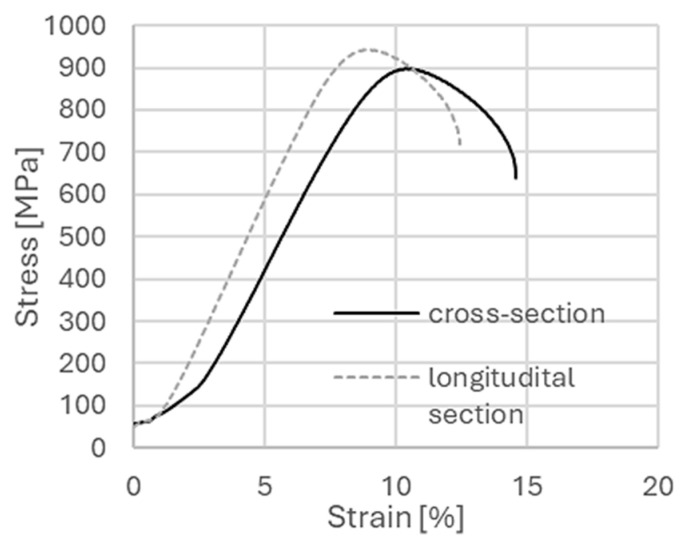
Tensile curves of micro specimens for titanium after the cumulative hydrostatic extrusion process for both test directions.

Figure 4 shows example tensile curves for both cross-sections for titanium after the cumulative hydrostatic extrusion process. A higher value of elongation in the transverse sample (where tensile forces act perpendicular to the axis of the extruded bar) and a higher strength in the longitudinal sample (where tensile forces act in line with the axis of the extruded bar) were observed. Table 3 summarizes the average values of ultimate tensile strength (UTS), yield strength (YS) and elongation (E) determined for both directions of the test.

**Figure 5:**
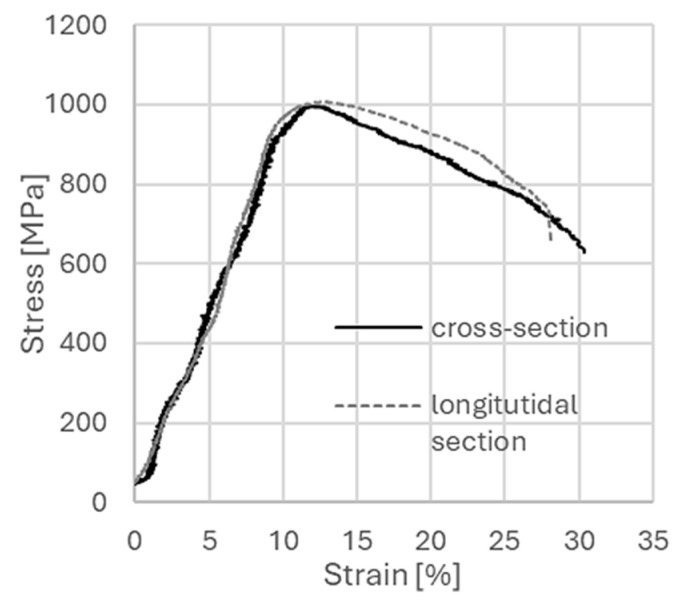
Tensile curves of micro specimens for titanium after the combination of HE + ECAP processes for both test directions.

The highest values for both yield stress and ultimate tensile strength were observed on the longitudinal section, with values about 10% and 4% higher, respectively. The elongation value was lower by about 35% on the longitudinal section. Differences in mechanical properties measured on both directions of the extruded bar are a characteristic feature in materials after the hydrostatic extrusion process. Earlier studies, carried out for titanium of grade 3 purity, showed even greater differences, despite similar summed strain rates [21]. Yield strength was 40% lower on the cross-section, and in the case of breaking strength, the differences were not so pronounced and reached about ~ 2–3%. Similarly, the elongation value was much lower on the longitudinal section. Similar tests after the hydrostatic extrusion process were conducted for CuCrZr alloy [26]. In this case, too, there were significant differences in the values of mechanical parameters measured in both directions, with both yield strength and ultimate tensile strength being significantly higher in the longitudinal direction by about 26% and 28%. The CuCrZr alloy is a good example of a material after a hydrostatic extrusion process which illustrates that anisotropy of mechanical properties is not always an undesirable phenomenon. It has been shown for this material that electrodes for spot welding processes having strong anisotropy have more than a 7-fold higher lifetime, both in comparison with commercial material and material highly comminuted after combination of severe plastic deformation ECAP + HE techniques [13]. Figure 5 shows an example of micro-sample tensile curves for titanium grade 2 after the combination of ECAP + HE with a total true strain of ε ~ 3.5.

In contrast to the data obtained for the hydrostatic extrusion process, in this case the two tensile curves for both the cross-section and the longitudinal section almost coincide. Clearly higher values of elongation to rupture up to 30% on the cross-sectional specimen are observed. Table 4 summarizes the mechanical test data for titanium after the combination of ECAP + HE with a total actual strain of ε ~ 3.5. Figure 5 shows an example of micro-sample tensile curves for titanium grade 2 after the combination of ECAP + HE with a total true strain of ε ~ 3.5. 

The UTS tensile strength values measured on the two cross-sections are practically no different from each other. It is also worth noting the low values of deviation from the average value. In the case of yield strength (YS), the average values are also very similar and the difference is at the level of about 1%. On the longitudinal section, a slightly lower value of average elongation is observed. The effect of the anisotropy of mechanical properties after the combination of the processes of squeezing through the ECAP angle channel and hydrostatic extrusion was practically eliminated. Compared to the process where only the hydrostatic extrusion method was used with the same total actual strain, significantly higher average values of mechanical properties are also observed with better ductility at the same time. Studies of the effect of combinations of the ECAP and HE techniques on the anisotropy of mechanical properties in materials have not been carried out to date. Typically, only properties on the longitudinal section were studied, using classical mechanical test specimens. A combination of these processes has so far been used for materials such as nickel, aluminum and aluminum alloys of the 5XXX series and copper or CuCrZr copper alloy [26,27,28]. In all cases, a significant increase in mechanical properties was found after the combination of ECAP and HE compared to the HE method alone, along with a strong homogenization of the microstructure.

### 3.2. Microstructure

**Figure 6:**
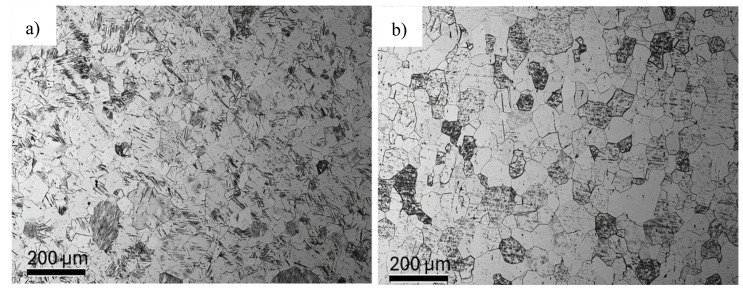
Microstructure of titanium in the initial state after the annealing process at 700 °C for 2 h: (**a**) cross-section and (**b**) longitudinal section.

Figure 6 shows the microstructure of titanium in the initial state after annealing at 700 °C for 2 h for the cross-section of the bar and the longitudinal section. 

**Figure 7:**
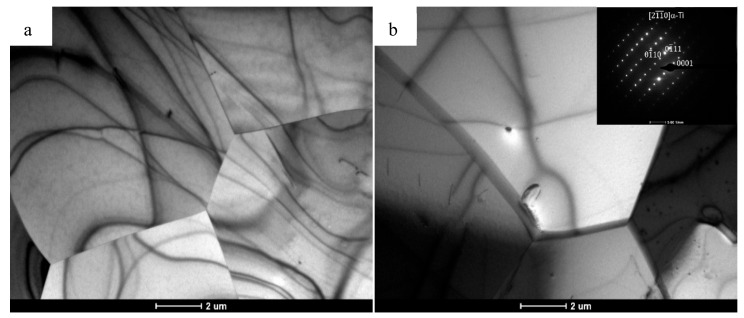
TEM/BF images of the microstructure obtained from a rod made of grade 2 titanium annealed at 700 °C for 2 h in air: (**a**) longitudinal cross-section and (**b**) cross-section along with SAED diffraction image from a single α-Ti grain.

The measured average grain size on the cross-section was deqp = 38 µm (±6) and deqt = 43 µm (±8) on the longitudinal section. On the basis of the microstructure images obtained, grain aspect ratios were determined by image analysis, which reached similar values for the cross-section αPD = 1.33 and for the longitudinal section αTD = 1.34. Figure 7 shows TEM/BF microstructure images for titanium in the initial state after the annealing process.

**Figure 8:**
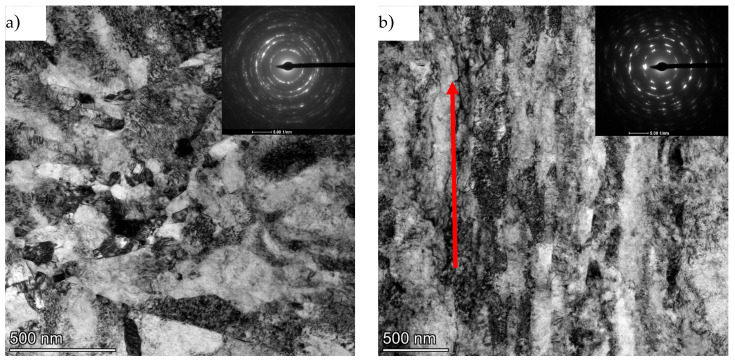
TEM/BF images of titanium microstructure after the hydrostatic extrusion process with total true strain ε ~ 3.5: (**a**) cross-section and (**b**) longitudinal section along with SAED diffraction images. The arrow indicates the direction of extrusion.

From the TEM/BF images, it can be seen that in both cases the microstructure consists of equiaxial grains. Most of the grains are almost free of structural defects, with only a minor number of dislocations. No pores or structural discontinuities were observed in the presented microstructure images. Typical artifacts of TEM imaging in bright-field mode, such as extinction contours, were also observed. A phase analysis was also carried out based on the indexing of SAED patterns by measuring the distance between the central and diffracted spots. These measurements were compared with the model diffraction pattern at the specific crystallographic orientation of the phase predicted as the solution. Hence, it is concluded that only an α-Ti phase with an hcp structure is present in the material. Based on the presented microstructure images on both cross-sections of the obtained rod, it was concluded that the material has a fully recrystallized equiaxial structure, which at the same time confirmed the appropriate choice of heat treatment parameters for the material. The material prepared in this way was a suitable isotropic model for further steps involving plastic processing by hydrostatic extrusion and ECAP. Figure 8 shows the microstructure of titanium in cross-section and longitudinal section after the hydrostatic extrusion process with a total true strain of ε ~ 3.5. A highly defected and refined microstructure is observed on the cross-section transverse to the extrusion direction. Locally, we can see aggregates of very fine, well-formed grains with sizes in the range of about ~ 100 nm. The strongly refined microstructure observed on the cross-section is evidenced by very clearly defined circles on the SAED diffraction patterns originating from multiple grain orientations. A completely different character of the microstructure is observed on the longitudinal section. The microstructure consists of defected, highly elongated grains arranged in bands aligned with the extrusion direction.

**Figure 9:**
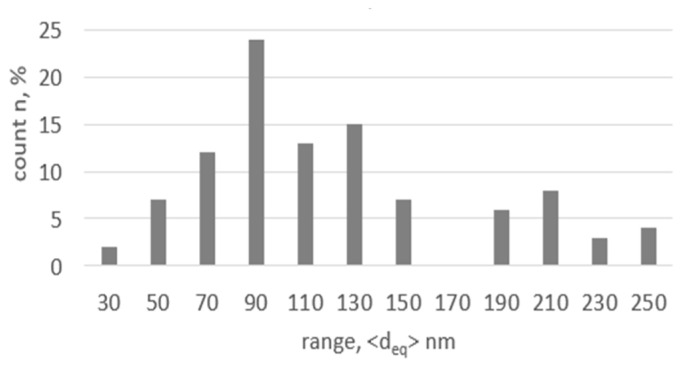
Grain size distribution on cross-section in titanium after the hydrostatic extrusion process.

The thickness of the bands corresponds to the size of the grains observed on the cross-section. Much less spread of crystallographic orientations is observed on the SAED image, with an almost point-like pattern indicating strong microstructural anisotropy, compared to the images of the transverse direction. The average size on the transverse section, as measured by image analysis, is deq = 123 nm (±56), with the coefficient of variation of the grain size distribution (Cvdeq) = 0.47. Figure 9 shows the grain size distribution measured after the hydrostatic extrusion process on the transverse section. The large fraction of grains with an average deq size of ~ 100 nm and the prominent fraction of grains with a deq size of more than 200 nm affect the high value of the coefficient of variation of distribution (Cvdeq), which indicates that the microstructure is not very homogeneous.

**Figure 10:**
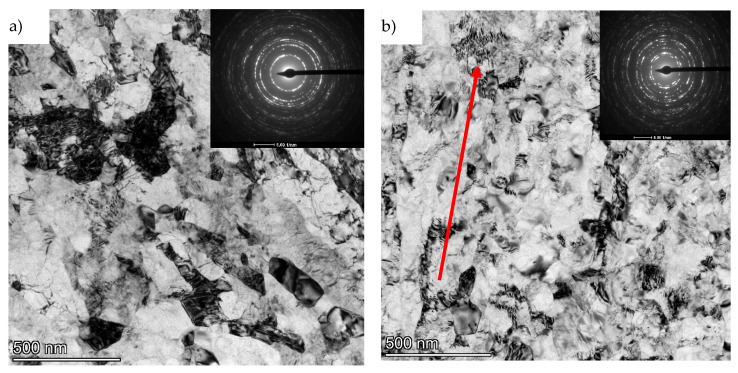
TEM/BF images of titanium microstructure after the combination of ECAP and HE processes with a combined true strain of ε ~ 3.5: (**a**) cross-section and (**b**) longitudinal section along with SAED diffraction images. The arrow indicates the direction of extrusion.

A similar character of the microstructure was observed on the longitudinal section for titanium of grade 3 purity, where the total true strain after the hydrostatic extrusion process was ε = 3.24 [29]. The thickness of the observed bands on the longitudinal section for grade 3 titanium was estimated by the authors to be between 60 and 600 nm. The observed SAED diffraction images were also similar in nature, where no clearly defined grain boundaries were observed with diffuse images. Figure 10 shows images of the microstructure after a combination of the processes of hydrostatic extrusion (HE) and equal channel angular pressing (ECAP) with a total true strain of ε ~ 3.5.

**Figure 11:**
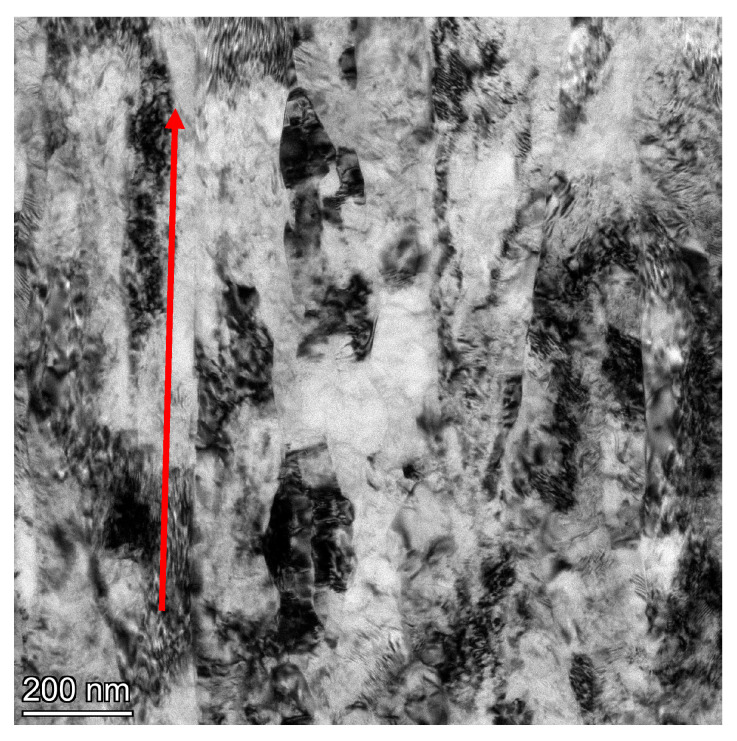
TEM/BF image of the titanium microstructure after the combination of the ECAP and HE processes with a total true strain of ε ~ 3.5 on the longitudinal section together The arrow indicates the direction of extrusion.

On the cross-section, we observe a defected microstructure with well-formed grains. This is also evidenced by the very distinct rings observed in the SAED images. The average grain size as measured by image analysis is deq = 74 nm (±20) with a coefficient of variation of grain size distribution (Cvdeq) = 0.25. The almost twofold lower value of Cvdeq compared to titanium after extrusion in this case indicates a much higher microstructural homogeneity. In contrast to the microstructure images presented for the longitudinal section, after the hydrostatic extrusion process, a well-developed grain microstructure is also observed on the longitudinal section after the combination of the ECAP and HE processes. SAED images in this case also show well-formed rings from multiple orientations. The average grain size on the longitudinal section is deq = 95 nm (±35) with the coefficient of variation of the grain size distribution (Cvdeq) = 0.35 and is similar in error to the value measured on the transverse section. The higher value of the coefficient of variation of the Cvdeq distribution and the slightly larger average grain size (deq) compared to the cross-section is due to the still locally present bands and grains oriented according to the extrusion direction; however, these bands are shorter and clearly interspersed with fine grains. An example of such an area is shown in Figure 11. 

**Figure 12:**
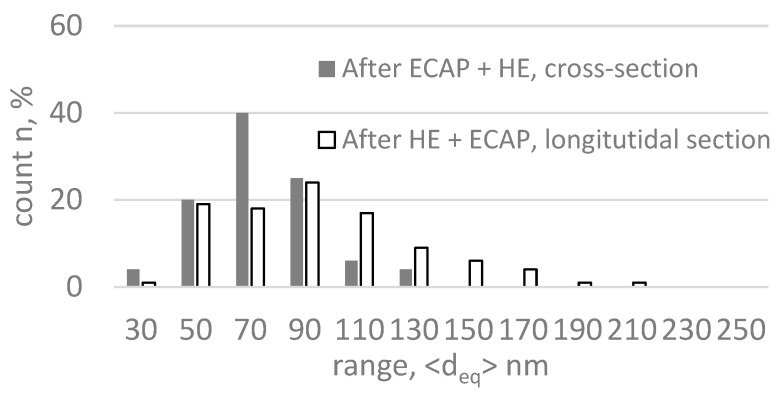
Grain size distribution on cross-section and longitudinal section in titanium after combination of ECAP and HE processes with total true strain ε ~ 3.5.

The slight differences observed in the two cross-sections are well illustrated by the grain size distributions shown in Figure 12. Despite the similar average grain size on both cross-sections, a larger fraction of grains above ~100 nm is observed on the longitudinal section, related to their elongation in the extrusion direction. The similar grain sizes and high degree of grain formation correspond well with the results of mechanical tests, where the tensile curves practically overlapped for both test directions. The higher degree of refinement of the microstructure and its better homogeneity after the combination of the ECAP and HE processes explain the significantly higher values of the mechanical properties compared to the material with the same total actual strain subjected only to the hydrostatic extrusion process. This applies both to yield strength (YS) and ultimate tensile strength (UTS) and also to elongation, which, due to the high homogeneity of the microstructure, is also significantly improved.

### 3.3. EBSD Analysis

**Figure 13:**
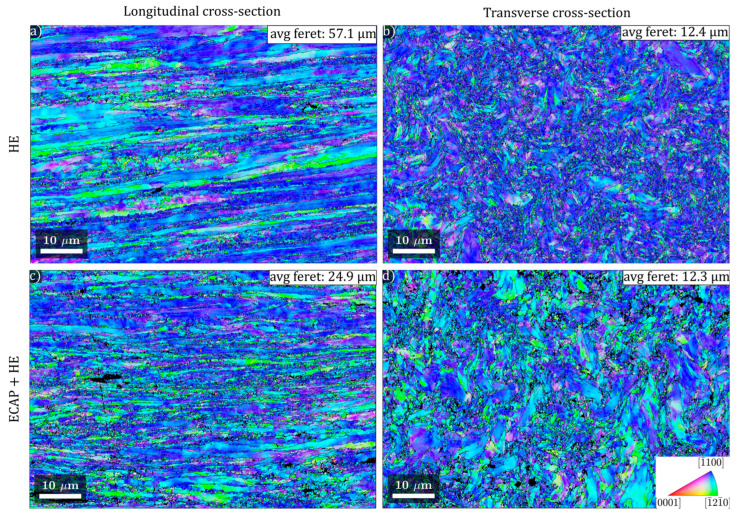
Inverse pole figure (IPF) maps of TiGr2 microstructure after hydrostatic extrusion (HE) (**a**,**b**) and combined ECAP + HE (**c**,**d**) processing. IPF coloring corresponds to the extrusion direction. HAGBs are indicated by black lines, and LAGBs are indicated by grey lines.

The microstructure of the processed titanium rods was investigated at a larger scale using the EBSD technique. Maps measuring 82 µm × 56 µm with a 100 nm step size were acquired from both longitudinal (long) and transverse (trans) cross-sections. It should be pointed out that the EBSD results presented here are complimentary to the results of the TEM microstructure analysis, registering the microstructure and heterogeneity at a much larger scale. The titanium microstructure after hydrostatic extrusion exhibits relatively high heterogeneity across both micro- and macro-scales. It can be described as bimodal, with a high density of grain boundaries. In the EBSD maps, grains appear highly elongated; therefore, the maximum Feret length was used for grain size characterization instead of the equivalent circle diameter. In the longitudinal cross-section (Figure 13a), grains are highly elongated in the extrusion direction, with lengths exceeding 80 µm and thicknesses ranging from 2 µm to 10 µm. Between the large grains, clusters of highly refined grains with sub-micron to micron sizes and more ellipsoidal shapes are observed. The grain boundary density is estimated by quantifying cumulative boundary segment lengths of a specific boundary type with respect to the total surface area of the analyzed EBSD map. After HE, a significant increase in grain boundary density is evident: 3.05 µm^−1^ for high-angle grain boundaries (HAGBs) and 3.96 µm^−1^ for low-angle grain boundaries (LAGBs), compared to the recrystallized state before extrusion (0.21 µm^−1^ and 0.03 µm^−1^, respectively). However, HAGB-enclosed grain regions are not well-defined. Despite the appearance of relatively distinct narrow bands, grain boundaries exhibit low connectivity; HAGBs frequently drop below the 15° misorientation threshold. This hampers typical grain size analysis via EBSD reconstruction; reconstructed grains often exceed 20 µm in thickness, exhibit high internal misorientation and contain many disconnected HAGBs. In contrast, the transverse cross-section shows a markedly different morphology after HE, characterized by ‘curly’ grains.

**Figure 14:**
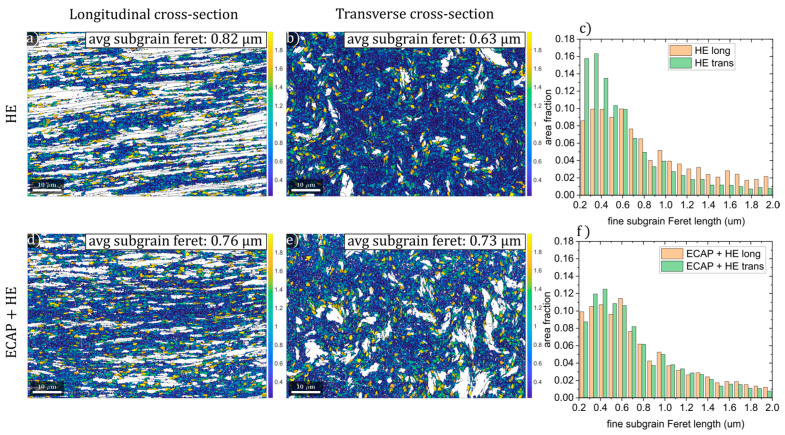
Fine subgrain analysis in titanium processed by hydrostatic extrusion (**a**–**c**) and in titanium processed by combined ECAP + HE (**d**–**f**). Maps present the subgrain size distribution of subgrains with lengths below a 2 µm threshold. White areas represent the coarse subgrain fraction (above 2 µm length).

The bimodal nature of the microstructure persists, with clusters of fine grains (sub-micron to micron scale) interspersed between larger grains (often >20 µm). As in the longitudinal section, many of the larger grains contain unconnected HAGBs and exhibit high internal misorientation, resulting in a lath-like appearance. Grain boundary densities are even higher than in the longitudinal section—4.97 µm^−1^ for HAGBs and 6.1 µm^−1^ for LAGBs. Comparing the longitudinal and transverse sections highlights strong anisotropy in the HE-processed microstructure, with average Feret lengths of 57.1 µm and 12.4 µm, respectively. In both sections, LAGB densities notably exceed those of HAGBs, with high LAGB populations observed in both refined grain clusters and large laths or bands. Following combined ECAP + HE processing (Figure 13c,d), the microstructure appears more refined than after HE alone. In the longitudinal section, elongated bands are narrower, and fine-grained clusters occupy a larger fraction of the area. Interestingly, the transverse section does not appear significantly more refined than after HE, and some larger grains with more homogeneous internal structure remain. These trends are reflected in the quantitative data; the average Feret length in the longitudinal section decreases markedly (from 57.1 µm to 24.9 µm), while it remains nearly unchanged in the transverse section (12.4 µm vs. 12.3 µm). The reduction in longitudinal grain size is accompanied by a slight increase in HAGB density (from 3.05 to 3.32 µm^−1^) and a decrease in LAGB density (from 3.96 to 3.14 µm^−1^). This may indicate that deformation energy stored during ECAP facilitated continuous dynamic recrystallization during subsequent HE, increasing misorientation at LAGBs and transforming them into HAGBs more effectively than with HE alone. To better understand the impact of ECAP + HE processing, a more detailed analysis of the fine microstructure was conducted. As mentioned earlier, standard grain size analysis based on a 15° misorientation threshold (below which LAGBs can be described as an arrangement of individual dislocations, and above which the grain boundaries become disordered regions in a crystal lattice) can yield unrealistically large grain sizes and make the results highly sensitive to EBSD scan area selection—potentially skewing average grain size values. To address this, an alternative reconstruction was performed using a 2° misorientation threshold to analyze subgrains. This excludes the lowest misorientation segments associated with crystal lattice bending and individual geometrically necessary dislocations and uses more clearly defined subgrain boundaries for grain reconstruction. Maps were then filtered to include only subgrains with Feret lengths below 2 µm. The resulting maps, with subgrains color-coded by size, along with corresponding size histograms, are shown in Figure 14. After HE, the differences between longitudinal and transverse sections are immediately apparent. In the longitudinal section, fine subgrains constitute only 53% of the microstructure, with an average Feret length of 0.82 µm. In contrast, the transverse section has 87% fine subgrains, with a smaller average Feret length of 0.63 µm. The subgrain size distribution (Figure 14c) in the longitudinal section is visibly shifted to the right (larger sizes), whereas in the transverse section the peak occurs at finer subgrain sizes. In contrast, samples processed via ECAP + HE show a much more balanced representation of fine subgrains: 69% in the longitudinal and 75% in the transverse section, with average lengths of 0.76 µm and 0.73 µm, respectively. Their size distributions are nearly identical (Figure 14f), indicating a more uniform microstructure. This clearly suggests that ECAP + HE processing leads to a more isotropic microstructure, which likely explains the more consistent mechanical behavior observed during tensile testing. In ECAP + HE samples, similar numbers of grain and subgrain boundaries are exposed to strain in both directions, resulting in a comparable density of obstacles to dislocation glide. In contrast, HE-processed samples experience strain in microstructures with vastly different boundary densities depending on the direction, leading to distinct dislocation environments. This may also influence grain boundary sliding [30,31], which may be associated with the enhanced plasticity of ultrafine-grained titanium.

**Figure 15:**
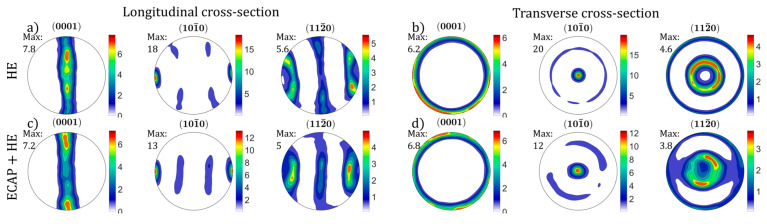
Pole figure plots after HE: (**a**,**b**) and combined ECAP+HE: (**c**,**d**), recalculated from the EBSD maps presented in Figure 13.

Figure 15 shows the pole figure plots related to the presented EBSD maps. The pole figures calculated from the EBSD orientation data indicate a strong axial texture typical for hcp metal products after axisymmetric plastic deformation process. In both cases, HE and combined ECAP + HE processing results in strong preference of ($10\bar{1}0$) poles alignment with the extrusion direction, and (0001) poles are distributed radially in plane perpendicular to the extrusion direction (Figure 15b,d). However as indicated by maximum intensities in the ($10\bar{1}0$) PF maximum along the extrusion direction, the texture after combined ECAP + HE processing is substantially softened, as evidenced by a decrease in the ($10\bar{1}0$) PF peak intensity from 20 multiples of random (rdm) to 12 rdm.

This result indicates a remarkable effect of the combined ECAP + HE on the reduction in texture anisotropy in comparison to HE processing. The decrease in ($10\bar{1}0$) PF peak intensity suggests that ECAP processing introduces a considerable number of crystallites with ($10\bar{1}0$) poles oriented away from the principal extrusion direction. As the ($10\bar{1}0$) crystal planes are involved in primary 〈a〉 dislocation slip in titanium, the softening of the texture can decrease the strain hardening rate and premature fracture. Moreover, it was shown previously that ($10\bar{1}0$) texture softening, specifically through plastic-forming processing with strain path change, can improve plasticity during subsequent forming, due to higher activation of c+a dislocation sliding in Ti6Al4V alloys [32].

## 4. Conclusions

The main findings of this study can be summarized as follows:

1. Application of ECAP prior to HE eliminated the anisotropy observed after HE alone, resulting in almost identical mechanical properties in longitudinal and transverse directions.
2. The ECAP + HE process produced significantly higher mechanical properties compared with HE alone: UTS ~ 1000 MPa, YS ~ 945 MPa and elongation ~ 25%.
3. TEM and EBSD analyses confirmed a much finer and more homogeneous grain structure after ECAP + HE (deq = 74–95 nm) compared to HE (deq = 123 nm with a bimodal distribution).
4. The coefficient of variation of grain size decreased markedly after ECAP + HE, indicating improved microstructural uniformity.
5. Balanced distributions of fine subgrains were obtained after ECAP + HE (69% longitudinal, 75% transverse) compared to those obtained following HE, which showed pronounced anisotropy.
6. Texture softening in combined ECAP + HE has positive effect on the plasticity by introducing spread in $\left(10\bar{1}0\right)$ planes distribution and preventing excessive dislocation pile-ups associated with strain hardening.

## Figures and Tables

**Table 1:** Chemical composition (wt.%) of the initial commercially pure titanium grade 2 used in this study (in accordance with ASTM B348-21).

Fe	C	N	H	O	Ti
0.110	0.031	0.01.	0.001	0.15	Balance

**Table 2:** Parameters of the two combinations of the plastic deformation processes of titanium.

**The process of cumulative hydrostatic extrusion**							
**Stage**	**d_0_ [mm]**	**d_f_** **[mm]**	**R**	**ε**	**ε_tot_**	**V** **[mm/s]**	**P_HE_** **[MPa]**
HEx1	29	16	3.29	1.19	1.19	9.4	911
HEx2	16	10	2.56	0.94	2.13	9.4	1014
HEx3	10	7	2.04	0.71	2.84	9.4	922
HEx4	7	5	1.96	0.67	3.51	9.4	874
**ECAP + HE combination**							
ECAPx1	15	15	-	0.66	0.66	-	-
ECAPx2	15	15	-	0.66	1.33	-	-
HEx1	15	10	2.25	0.81	2.14	7.5	673
HEx2	10	7	2.04	0.71	2.85	7.5	713
HEx3	7	5	1.96	0.67	3.52	7.5	802

Where d_0_—initial diameter, d_f_—final diameter, R—reduction (d02/df2), ε—true strain, ε_tot_—total true strain, V—linear extrusion velocity and P_HE_—extrusion pressure.

**Table 3:** Average values of mechanical properties after the process of hydrostatic extrusion of titanium, measured on both directions of the extruded rod.

Test Direction	UTS [MPa]	YS [MPa]	E [%]
Cross-section	901 ± 8	787 ± 12	18 ± 3
Longitudinal section	932 ± 11	857 ± 10	12 ± 1.2

**Table 4:** Average values of mechanical properties after the combination of ECAP + HE titanium processes, measured on both directions of the extruded bar.

Test Direction	UTS [MPa]	YS [MPa]	E [%]
Cross-section	1001 ± 5	943 ± 16	25 ± 3
Longitudinal section	1008 ± 7	952 ± 18	21± 2

## Data Availability

The original contributions presented in this study are included in the article. Further inquiries can be directed to the corresponding author.

## Abbreviations

The following abbreviations are used in this manuscript:

ECAP	Equal-channel angular pressing
HE	Hydrostatic extrusion
TEM	Transmission electron microscopy
SEM	Scanning electron microscopy
EBSD	Electron backscatter diffraction
SAED	Selected area electron diffraction
UTS	Ultimate tensile strength
YS	Yield strength
E	Elongation
HAGB	High-angle grain boundary
LAGB	Low-angle grain boundary
UFG	Ultrafine-grained
HPT	High pressure torsion
SPT	Small punch test
